# The Influence of the Intergranular Superdisintegrant Performance on New Drotaverine Orodispersible Tablet Formulations

**DOI:** 10.3390/pharmaceutics15082147

**Published:** 2023-08-16

**Authors:** Robert-Alexandru Vlad, Cezara Pintea, Diana-Andreea Chirteș, Paula Antonoaea, Emöke Margit Rédai, Nicoleta Todoran, Magdalena Bîrsan, Adriana Ciurba

**Affiliations:** 1 Pharmaceutical Technology and Cosmetology Department, Faculty of Pharmacy, George Emil Palade University of Medicine, Pharmacy, Science and Technology of Targu Mures, 540142 Targu Mures, Romania; 2 Faculty of Pharmacy, George Emil Palade University of Medicine, Pharmacy, Science and Technology of Targu Mures, 540142 Targu Mures, Romania; 3 Targu Mures Clinical County Hospital, 540072 Targu Mures, Romania; 4 Drug Industry and Pharmaceutical Biotechnology Department, Faculty of Pharmacy, “Grigore T. Popa” University of Medicine and Pharmacy from Iasi, 700115 Iasi, Romania

**Keywords:** intragranular superdisintegrant, intergranular disintegrant, fast-dissolving granules, drotaverine orodispersible tablets, pharmacotechnical evaluation

## Abstract

The main objective of this study consists in establishing the influence of the intergranular superdisintegrant on the specific properties of drotaverine hydrochloride fast-dissolving granules (DROT-FDGs) and orodispersible tablets (DROT-ODTs). The orodispersible tablets were obtained by the compression of the FDGs and excipient mixture with an eccentric tableting machine. To develop DROT-ODTs, two types of superdisintegrant excipients in different concentrations (water-soluble soy polysaccharides (SSP) (1%, 5%) and water-insoluble soy polysaccharides—Emcosoy^®^ STS IP (EMCS) (1%, 3%, 5%)) were used, resulting in five formulations (D1–D5). The DROT-FDGs and the DROT-ODTs were subjected to pharmacotechnical and analytical evaluation. All the orodispersible tablets obtained respect the quality requirements in terms of friability (less than 1%), crushing strength (ranging between 52 N for D2 and 125.5 N for D3), and disintegration time (<180 s). The in vitro release of drotaverine from ODTs showed that all formulations presented amounts of active substance released greater than 85% at 10 min. The main objective, developing 30 mg DROT-ODTs for children aged between 6 and 12 years by incorporating the API in FDGs, was successfully achieved.

## 1. Introduction

In the last decade, the pharmaceutical industry has turned its attention not only to well-known pathologies with impressive numbers of patients but also to tighter categories of people suffering from less life-threatening conditions. Thus, innovative formulations such as orodispersible tablets (ODTs) were developed to ease the administration process for pediatric and geriatric patients and to assure the intake of active substances in psychiatric disorders. They can also be prescribed for ordinary use, but are most beneficial in case of swallowing difficulties (dysphagia). ODTs are developed in single-unit dosage forms destined for oral administration, which disintegrate or dissolve in contact with saliva, without requiring water for swallowing [1,2]. The disintegration process is limited by the *European Pharmacopeia 10th Edition* (*Ph. Eur. 10*) at 3 min, but usually finishes in the first minute, resulting in faster absorption, increased bioavailability, and fewer side effects [3]. In addition to the advantages aforementioned, ODTs improve patient compliance by offering fast to immediate effects and by masking the unpleasant taste that some drugs possess. They offer simple transport, are favorable in terms of dosage, and limit the risk of choking during oral administration of classical tablets or capsules [1,3].

For manufacturing ODTs, multiple technologies can be used: some conventional (direct compression, freeze-drying, tablet molding, spray-drying, sublimation) and some patented technologies highlighted in Table 1 (Zydis **^®^**, Orasolv**^®^**, Durasolv**^®^**) [4,5].

Direct compression is the easiest and most cost-effective of all methods, using a conventional tablet press for manufacturing, while the disintegrating process is assured by disintegrants or superdisintegrant excipients. Additionally, wet granulation is a widespread technology applied for powders in order to elevate the level of flowability, easing the compression procedure [6,7,8]. The excipients used for formulating ODTs are cautiously selected corresponding to the manufacturing process. For these kinds of preparations, the most important type of excipients are the disintegration facilitators that liberate the API and make it susceptible to further absorption and metabolization [9]. Along with these, a large number of substances can be used as excipients (Table 2) to perform as dilution agents, taste correctors, lubricants (antiadherents and glidants), agglutinants, and sweeteners [10,11].

The choice to use soybean polysaccharides in the formulation process comes after intensive research, highlighting the superdisintegrant action of this compound [12]. Both water-soluble soy polysaccharides (SSP) and water-insoluble soy polysaccharides (Emcosoy**^®^** STS IP) have been proven to provide an admirable disintegration capacity and previously included in tablets designed for sublingual use [13,14].

**Figure 1:**
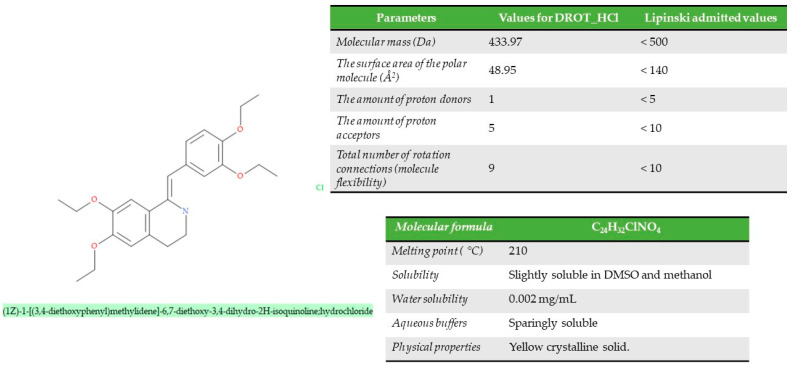
Molecular, structural, and physical–chemical properties of the DROT-HCl.

From a structural point of view, drotaverine (1Z)-1-[(3,4-diethoxyphenyl)methylidene]-6,7 diethoxy-3,4-dihydro-2H-isoquinoline (Figure 1) is a benzylisoquinoline derivative, with the molecular formula C_24_H_31_NO_4_ and a molecular weight of 397.5 g/mol (433.97 Da). It is presented in the form of a yellow, odorless powder with a strong bitter taste. The active pharmaceutical ingredient (API) is slightly soluble in water, and the aqueous solution has an acidic reaction with a pKa of 7.11 and a logP of 5.35. In therapy, it is used in the form of drotaverine hydrochloride, a salt obtained with chlorohydric acid [2,15,16]. Respecting the Lipinski Rule of Five, the structural and molecular data have to be less than the values stipulated in Figure 1. The API can be administrated orally, intravenously, or intramuscularly, and the method of administration not generating a significant change in pharmacological parameters (half-life, clearance, apparent distribution volume).

The absorption of DROT-HCl is complete and rapid both after parenteral and oral administration in rats [17]. Regarding distribution, the selected API is known for its strong binding to plasma proteins (95–98%), notably to albumin, but to beta and gamma globulins as well. DROT-HCl is rapidly metabolized at hepatic level by O-deethylation [18], and it is excreted through urine and feces as highlighted in human studies [19]. In a study conducted on rats, it has been shown that the active ingredient can be eliminated into the bile-producing conjugated metabolites, among which 4′desethyl-drotaveraldine is predominant [20].

DROT-HCl is an intense musculotropic antispasmodic drug, whose action can be explained by its capacity to inhibit the activity of the phosphodiesterase IV enzyme, which increases cAMP resulting in lower intracellular calcium levels [21]. As a muscle relaxant, its effects are focused mainly on the smooth muscles found in the gastrointestinal and urinary tracts. It is used for alleviating pain associated with irritable bowel syndrome, biliary colics, and renal colics, along with gynecological conditions such as dysmenorrhea [22]. Considering that it does not cross the placental barrier, DROT-HCl is used with great results in the dilatation stage of labor for shortening its length [18]. Furthermore, an enormous benefit is found in the lack of anticholinergic side effects and the infrequent adverse reactions [21].

Croscarmellose sodium was also used with great results as a superdisintegrant for breaking apart the tablet and assuring the release of API [23]. The preparation methods can vary from using solid mixture techniques [16] to applying melt granulation methods [2] or manufacturing ODTs with coprocessed mixtures [24]. What is certain is that ODTs are emerging as a fitting formulation for increasingly more APIs and the pharmaceutical industry will continue to benefit from its many advantages.

Until now, few articles had the same (final) aim of developing orodispersible tablets (ODTs) with DROT-HCl and none included the disintegrants outlined in this study (insoluble/soluble in water soy polysaccharides). Furthermore, a small number of research articles have highlighted the importance of soy polysaccharides as disintegrants [12,13,14]. Another asset of this study is its use of water-soluble soy polysaccharides, which were not mentioned in any article for their disintegrant property or in any article describing their influence regarding the disintegration behavior.

The main objective of this study is the development of ODTs having DROT-HCl as an API (with 30 mg DROT-HCl considering the age range 6–12 years) using drotaverine fast-dissolving granules (DROT-FDGs) as an intermediate pharmaceutical formulation, and considering that, until now, the available pharmaceutical formulations (with DROT-HCl) on the market in Romania are tablets (Antispasmin, No-Spa, No-Spa forte, Spasmocalm), coated tablets (No-Spa Forte), capsules (Spaverin 40 and 80 mg), and injections (No-Spa 40 mg/2 mL—injectable solution) [25].

## 2. Material and Methods

### 2.1. Granules Preparation Steps

The FDGs were obtained through the wet granulation technique (composition in Table 3). In this regard, the following ingredients were used: 98% purity drotaverine hydrochloride—DROT-HCl (Rich Pharmachem, Maharashtra, India); Vivasol**^®^** GF Sodium Croscarmellose—CCSNa (JRS Pharma, Rosenberg, Germany); lactose—LCTS, Lactopress**^®^** Spray Dried (DFE Pharma, Goch, Germany); polyvinylpyrrolidone—PVP, Kollidon**^®^** 25 (BASF, Ludwigshafen am Rhein, Germany); and ethanol 96% *v*/*v* (Chimreactiv SRL, Bucharest, Romania).

The previously mentioned powders were mixed in a mortar respecting the following order: LCTS, CCSNa, PVP, DROT-HCl, and ground for three minutes to obtain a homogenous powder. The last ingredient added was ethanol 96% (*v*/*v*) which served as an agglutinant. The malleable mixture was sifted through an 800 µm sieve, and the granules obtained were dried at room temperature for 24 h after which the DROT-FDGs were incorporated in ODTs. The granules were evaluated in terms of color homogeneity and shape.

### 2.2. DROT-ODTs Manufacturing Steps and General Appearance Evaluation

To obtain the DROT-ODTs, the following ingredients were used (Table 4): DROT-FDGs; sweetener—sucralose (Myprotein, Bucharest, Romania); lubricant—sodium stearyl fumarate, Pruv**^®^** (JRS Pharma, Rosenberg, Germany); disintegrants—Emcosoy**^®^** STS IP (EMCS) (JRS Pharma, Rosenberg, Germany) and soluble soy polysaccharides (SSP) (Creative enzymes, San Diego, CA, USA); flavor agent—banana flavor (Elision Pharma, Vadodara, India); and filler—lactose (Lactopress**^®^**, (LCT) (DFE Pharma, Kaponga, New Zealand)). The powders and the DROT-FDG were added in a mortar respecting the characteristic rule of mixing and blended carefully to maintain the mixture granulometry until a homogenous mixture was obtained. The mixture was compressed with the help of an eccentric tableting machine and 12 mm punches. The DROT-ODTs were evaluated in terms of shape and color homogeneity, in addition to the well-known variables that are stipulated in the literature and the in-force pharmacopeias.

### 2.3. DROT-FDG Evaluation

The developed DROT-FDGs were evaluated in terms of particle size distribution, relative homogeneity index, particles <160 µm, DROT-HCl content, and dissolution behavior. The particle size distribution and relative homogeneity index tests are described extensively in studies where the SeDeM/SeDeM-ODT expert systems are used to develop conventional or orodispersible tablets [26,27,28,29,30,31,32].

#### 2.3.1. Particle Size Distribution

The particle size distribution was achieved by sifting the obtained DROT-FDGs for 10 min using a pre-established set of sieves arranged in descending order considering the mesh size of the sieve (4000, 2500, 800, 315, 200, and 160 µm). For each range, the average mesh was calculated as the dimension of the previous mesh with the mesh where the granules did not trespass divided by two, as in Table 5 [33]. The amount of granules that remained on each sieve was weighed using a four-decimal scale (Kern, Kern, Germany), and the average particle size was determined altogether with the granulometric curve.

#### 2.3.2. Relative Homogeneity Index

The relative homogeneity index was achieved considering the same experimental method described in Section 2.3.1 and calculated using Equation (1):

(1) $$I\theta =\frac{\mathrm{Fm}}{100+\;\Delta \mathrm{Fm}}$$

where

Iθ—relative homogeneity index;
Fm—percentage of particles in the majority range;
Fmn—percentage of particles in the superior majority range [29,30,31,32,33].

The obtained value regarding the relative homogeneity index was converted into a radius value by applying Equation (2).

r_Iθ_ = 500 × Iθ (2)

where

r_Iθ_—relative homogeneity index converted into radius [33].

#### 2.3.3. Particles < 160 µm

Particles < 160 µm were evaluated through the same experiment as described in Section 2.3.2. To convert the obtained values into radius, Equation (3) was used:

10 − (z/5)(3)

z—% (m/m) of particles < 160 µm [31,32,33].

#### 2.3.4. DROT-HCl Content

The DROT-HCl content was evaluated spectrophotometrically using a Shimadzu spectrophotometer (Mettler Toledo, Columbus, OH, USA). To establish the amount of DROT-HCl, a stock solution with a concentration of 1 mg/mL was prepared that was diluted to obtain the following concentrations: 1, 2.5, 5, 10, 25, 50, and 100 µg/mL. A mass of granules corresponding to 30 mg DROT-HCl was weighed and dispersed into an HCl solution, pH = 1.2 (7 mL of concentrated HCl (Lachner, Továrni, Czech Republic) and distilled water until 1000 mL) that mimics the stomach’s pH. The same experiment was conducted in the case of the DROT-ODTs. The ODTs were ground in a mortar, brought into a 25 mL volumetric flask, and dispersed in the HCl solution with a pH of 1.2. A dilution of 100 was applied and the concentration of the filtered sample (via a 0.45 µm Millipore filter) was measured spectrophotometrically by the means of a UV-1800 Shimadzu Spectrophotometer (Mettler Toledo, Columbus, OH, USA) at a specific wavelength λ = 353 nm. The experiment was conducted in triplicate.

#### 2.3.5. Dissolution

The dissolution was performed on both granules and orodispersible tablets. In this regard, both paddle and basket methods were used for granules to establish which method would be more convenient for future use. The dissolution behavior was evaluated in a Biobase TFUT-3 tester (Tablet Four-Usage Tester, Biobase, Jinan, China) using a 900 mL volume of HCl solution, pH = 1.2 (with the following composition: 7 mL of concentrated HCl, 2 g of NaCl, and ultrapure water until 1000 mL), thermostated at 37 ± 0.5 °C with 50 rpm. For the DROT-ODTs, the paddles were used whilst the other parameters were not changed. To establish the amounts of API released, the same UV spectrophotometric method was used as described in Section 2.3.4. The experiment was conducted in triplicate and the results reported as average ± SD.

### 2.4. DROT-ODT Evaluation

#### 2.4.1. Organoleptic Properties

The DROT-ODTs were evaluated in terms of appearance and uniformity of color.

#### 2.4.2. Dimensional Parameters and Uniformity of Mass

In this subsection, the radius, diameter, thickness, and uniformity of mass are described as average ± SD values.

##### Uniformity of Mass

This parameter was evaluated considering the *Ph. Eur. 10* stipulations [34]. Twenty tablets were weighed on an analytical scale (Kern, Kern, Germany), and their average mass, standard deviation, and percentage deviation were calculated. The admitted percentage deviation for each tablet from the average mass was set to 5% considering the mass of a single dose unit.

##### Diameter, Thickness, and Radius

These dimensional parameters were evaluated with the help of a digital caliper (Yuzuki, India). In this regard, 10 tablets from each formulation were measured, and their average diameter/thickness/radius was calculated ± SD.

#### 2.4.3. Mechanical Characteristics

##### DROT-ODT Resistance to Crushing (Crushing Strength)

During this test, the force needed to disrupt the tablets by crushing was evaluated. Ten tablets from each formulation were placed between the jaws of the Biobase TFUT-3 tester (Tablet Four-Usage Tester, Biobase, Jinan, China). The results are expressed as the mean values ± SD [33].

##### Friability

To further characterize important mechanical properties, another parameter that has to be evaluated is friability. Thirteen tablets from each formulation were introduced in a drum and subjected to 25 ± 1 rpm for four minutes. The same apparatus as described in the section titled DROT-ODT Resistance to Crushing (Crushing Strength) was used. The maximum value considered for this parameter was 1% [33].

##### Crushing Strength–Friability Ratio (CSFR)

CSFR is an index used to measure tablet quality employing two previously calculated parameters (tablet crushing strength and friability). This physical strength measurement was calculated by dividing the crushing strength by friability (Equation (4)). The effect produced by the different types and amounts of disintegrant on the tablet’s mechanical strength can be evaluated by referring to the CSFR value of the tablets. Usually, a higher CSFR value implies better mechanical properties of the evaluated tablet [35,36,37].

CSFR = Resistance to Crushing/Friability(4)

##### Tensile Strength (Ts)

Another physical strength parameter, tensile strength (Ts), can be calculated by knowing the tablet’s diameter, thickness, and resistance to crushing. With this aim, Equation (5) was used:

Ts = 2 × F/π × d × h(5)

where

Ts = tensile strength (MPa);
F = resistance to crushing (N);
d = DROT-ODT diameter (m);
h = DROT-ODT thickness (m).

The results are reported as the mean ± SD for each of the five proposed formulations [38].

#### 2.4.4. pH

The pH was evaluated for three DROT-ODTs from each formulation. A DROT-ODT was dispersed in 20 mL of ultrapure water followed by the filtration of the solution through a 0.45 µm Millipore filter. The filtrate pH was measured with a pH meter (pH Check, TFA Dostmann, Wertheim am Main, Germany). The results are expressed as the average ± SD.

#### 2.4.5. Wetting Time

To evaluate the wetting time, filter papers were wetted with a methylene blue solution of 1% and placed on a Petri dish. On the paper filter, a tablet is placed and the time needed for the tablet to soak was measured (seconds—s). For each formulation, three tablets were evaluated and the results are expressed as the average ± SD [33].

#### 2.4.6. Disintegration Test (Behavior)

The disintegration time was evaluated with the help of the Biobase TFUT-3 tester (Tablet Four-Usage Tester, Biobase, Jinan, China). In the open-ended transparent tubes of the basket-rack assembly, 6 ODTs from each formulation were introduced separately in thermostated phosphate buffer (pH = 6.8), with a temperature of 37 ± 1 °C, and covered with a plastic porous disc. The device oscillated with a constant frequency rate that ranged between 28 and 32 cycles/min. The average disintegration time for six tablets from each formulation was assayed and the results highlighted as mean ± SD considering that the maximum admitted disintegration value is 180 s (*Ph. Eur.*) and 30 s (*United States Pharmacopoeia*—USP 44) [34,35,36,37,38].

#### 2.4.7. DROT-HCl Content

Ten tablets of each formulation were powdered and 0.5 g of the mixture weighed directly in a 25 mL volumetric flask. The powder was dispersed in the dissolution media (pH = 1.2) at 25 mL and subsequently diluted 100 times. The UV-Vis spectrum of the analyzed API was traced and the DROT-HCl content in the solution was evaluated as described in Section 2.3.4; the results are outlined as mean dose ± SD.

#### 2.4.8. Dissolution

For both FDGs and ODTs, the dissolution experiment is described in Section 2.3.4.

#### 2.4.9. Statistical Analysis—One-Way ANOVA

For all the evaluated parameters, a statistical evaluation using Brown–Forsythe ANOVA and Welch one-way ANOVA tests was performed with GraphPad Prism 9 software (Dotmatics, Boston, MA, USA). The results are represented as mean ± SD. The significance level was set to 0.05 (*p*) with the *p* values presented as numbers in the Results and Discussion sections with the following levels of significance:

- (*p* > 0.05), ns—not significant;
- (*p* ≤ 0.05);
- (*p* ≤ 0.01);
- (*p* ≤ 0.001);
- (*p* ≤ 0.0001).

## 3. Results

### 3.1. FDGs General Characterization

Considering our aim, the amount of granules that contain DROT-HCl (30 mg) was established using a UV-Vis spectrophotometric method in which the average amount of granules was found to be 0.3816 g. Yellow DROT-FDGs were obtained with irregular shapes (Figure S1) that were eventually evaluated in terms of homogeneity, content (API assay), and dissolution, using both the paddle and basket methods.

### 3.2. DROT-FDG Evaluation

The DROT-FDGs prepared were used as an intermediate pharmaceutical formulation, but they were also evaluated in order to correspond to the pre-established compendial and literature requirements.

#### 3.2.1. Particle Size Distribution

**Figure 2:**
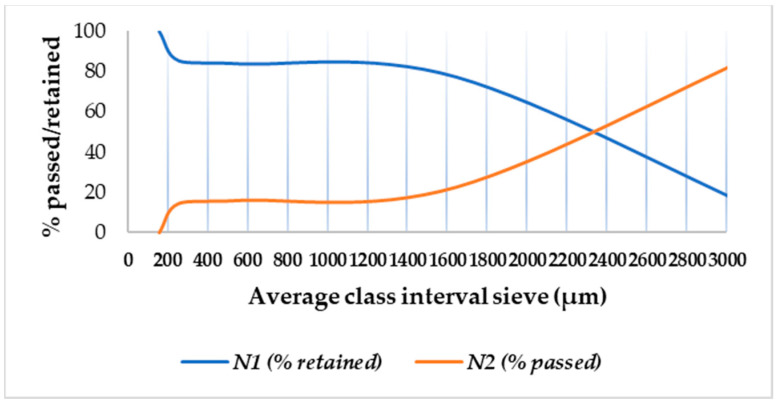
Granule cumulative frequency curve.

The cumulative results regarding the particle size distribution are provided in Table 5, and the frequency curve is shown in Figure 2.

**Figure 3:**
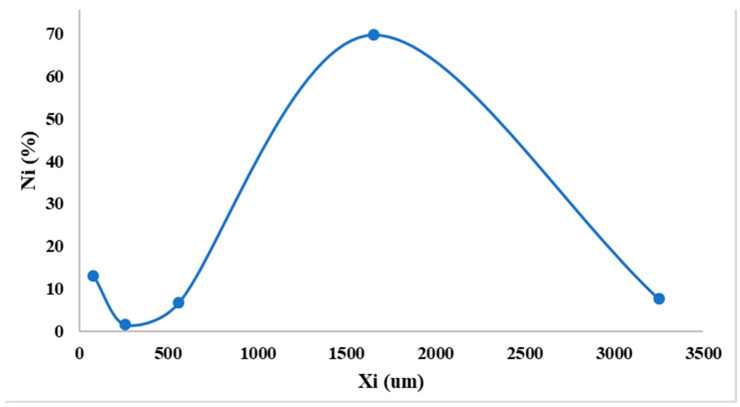
Granulometric curve.

It can be observed in Figure 3 that the developed DROT-FDGs are highlighting a Gaussian distribution with a maximum peak at 1600 µm; furthermore, it can be noticed that another peak should be taken into consideration, the one at the lower value, 100 µm, which is the lower limit regarding the granules dimension parameter. The FDGs dimension at 50% is 2.32 mm [37,38,39,40,41,42].

#### 3.2.2. Relative Homogeneity Index

The data used to evaluate the relative homogeneity index can be retrieved from Table 6. The index was calculated and the radius value obtained was maximum (10), which implies that the DROT-FDGs are respecting the dimension homogeneity. In the study conducted by Khan, in which ribavirin orodispersible tablets were developed, a granular mixture was analyzed with the SeDeM-ODT expert system. The results regarding the relative homogeneity index highlighted a lower value for the powder/granule blend (Iθ = 6.5) in comparison to this study, where the maximum value was obtained [33].

#### 3.2.3. Particles < 160 µm (0.16 mm)

The radius value for this parameter was 9.996, close to the maximum value (10). This parameter can show that granules with a dimension higher than 0.16 mm were obtained, respecting the dimensional criteria proposed by other studies reported in the literature and by the in-force pharmacopeial requirements. Our results cannot be compared to other studies because during DROT-FDG development, the dust was removed because it was considered a part of the manufacturing process. Furthermore, the size of the mesh used in this study was larger, which considered the pharmaceutical formulation (granules) that was developed [33].

#### 3.2.4. DROT-HCl Content

The DROT-HCl content from the FDGs was determined through a spectrophotometric method for which the linearity was evaluated in the concentration range 1.00–100.00 µg/mL (Figure S2a,b). The results outlined an average concentration of 28.35 ± 1.3 mg of DROT-HCl, which is close to the pre-established amount of 30 mg, additionally respecting the *Ph. Eur.* requirements regarding the uniformity of content (±15% of the declared amount).

#### 3.2.5. Dissolution

**Figure 4:**
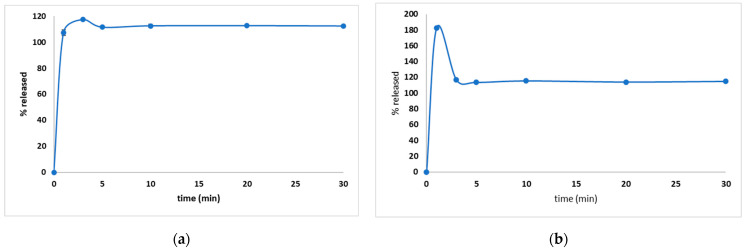
The dissolution behavior for both paddle (**a**) and basket (**b**) dissolution tests used to evaluate the amount of API released from the DROT-FDGs.

Even though most of the pharmacopoeias recommend the use of a basket to evaluate granules, in this study both basket and paddle dissolution tests were used to establish which is the most suitable for the prepared FDGs. Considering the composition of FDGs, a fast dissolution was expected, so the risk of granules-floating was eliminated. An immediate release was observed in both cases (Figure 4a,b), with a special mention that in the case of the basket method (Figure 4b), the API concentration was increased as a result of an agglomeration, but the concentrations decreased to the maximum amount included in the granules (close to 100%) as soon as the API dispersed homogenously in the dissolution media. Both methods can be useful whilst evaluating the dissolution behavior, noting that the paddle method gave constant concentrations during the experiment, whilst in the case of the dissolution test with a basket, the concentration after 3 min was more accurate and characteristic for evaluating the dissolution behavior.

### 3.3. DROT-ODT Evaluation

The DROT-ODTs were evaluated in terms of appearance, dimensional parameters, uniformity of mass, mechanical characterization, wetting time, disintegration time, DROT-ODT content, and dissolution behavior.

#### 3.3.1. DROT-ODTs Appearance

**Figure 5:**
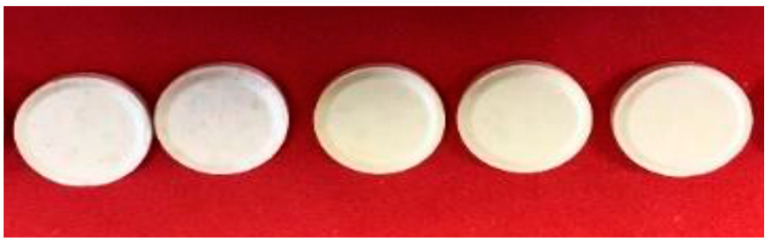
DROT-ODTs appearance.

The developed DROT-ODTs tend to be slightly (pale) yellow as a result of the presence of the API, the color is dispersed through the tablet uniformly and the edges are intact; the tablets have a banana smell as a result of the presence of the flavoring agent (Figure 5). The DROT-ODTs are displaced as flattened cylinders with a convex surface.

#### 3.3.2. Dimensional Parameters and Uniformity of Mass

##### Uniformity of Mass

**Figure 6:**
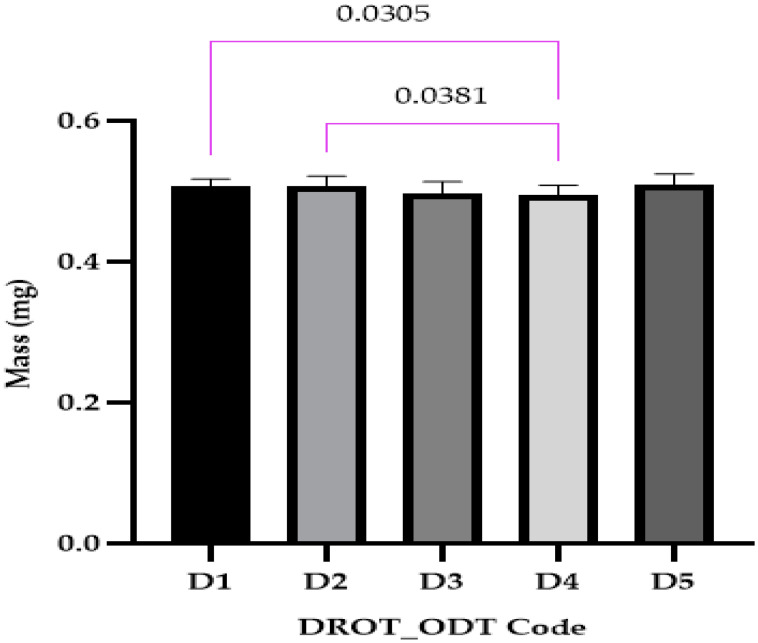
Uniformity of mass (average ± SD) of the developed DROT-ODTs Brown–Forsythe and Welch ANOVA test (multiple comparisons); significance level (*p* < 0.05). D1 (1% EMCS), D2 (5% EMCS), D3 (1% SSP), D4 (5% SSP), and D5 (3% EMCS) All unmarked comparisons correspond to nonsignificant differences (*p* > 0.05).

The DROT-ODTs mass results were close to 0.5000 g for all five formulations. The statistical analysis (Figure 6) revealed that two significant statistical differences were recorded between D1 and D4, where different superdisintegrants in different concentrations were used: 1% EMCS for D1 and 5% SSP for D4, and D2 (5% EMCS) and D4. It can be concluded that the type and the concentration of the disintegrant can produce differences that are statistically significant.

In the study conducted by Kuralla et al., DROT-ODTs were developed that contained different amounts of CCNa as a disintegrant and mannitol as a sweetener. The uniformity of mass ranged between 265 ± 1.05 mg for PF1 where 2% CCNa and no sweetener were used and 401 ± 1.54 mg, close to the values proposed in this study where the mass varied as a result of different amounts of ingredients used and the presence or absence of some excipients [2].

##### Diameter, Thickness, and Radius

**Figure 7:**
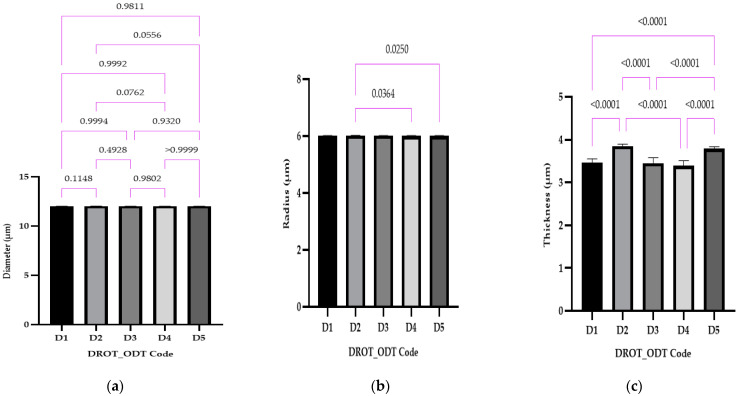
The DROT-ODTs diameter (**a**), radius value (**b**), and thickness (**c**) (average ± SD) of the developed DROT-ODTs Brown–Forsythe and Welch ANOVA test (multiple comparisons); significance level (*p* < 0.05). D1 (1% EMCS), D2 (5% EMCS), D3 (1% SSP), D4 (5% SSP) and D5 (3% EMCS). All unmarked comparisons correspond to nonsignificant differences (*p* > 0.05).

The statistical evaluation and the results obtained regarding the dimensional parameters can be retrieved from Figure 7: (a) diameter, (b) radius, and (c) thickness.

In the case of the diameter, the results were close to the pre-established value of 12 mm and no statistical differences were recorded (Figure 7a). For the radius, in two cases, statistical differences were found between D2 vs. D4, where different types of disintegrants were used—EMCS and SPP in the same concentration, and D2 vs. D5, where the same disintegrant was used (EMCS) in different concentrations (5% for D2 and 3% for D5) (Figure 7b).

Considering the thickness, Figure 7c, the following statistical differences were registered: D1 vs. D2 and D1 vs. D5 (as a result of different amounts of EMCS used 1% (D1), 3% (D5), and 5% (D3)) same concentration (5%), different superdisintegrant in the case of D2 vs. D4, and as a cumulation of factors for D2 (5% EMCS) vs. D4 (5% SSP), D3 (1% SSP) vs. D5 (3% EMCS), and D4 (5% SSP) vs. D5 (3% EMCS) where different superdisintegrants in varied concentration were used.

In the study conducted by Kuralla et al., 10 mm punches were used to develop DROT-ODTs; higher values regarding the thickness were obtained (>4 mm) in comparison to our study, where values <4 mm were obtained [2]. The different excipients used, the method the DROT-HCl was applied for incorporation, the weight of a DROT-ODT, and the different punches all might produce differences regarding thickness of the ODTs [2,24].

#### 3.3.3. Mechanical Characterization

Four tests were used to establish the ODT’s mechanical characteristics: resistance to crushing, friability, crushing strength–friability ratio, and tensile strength. These parameters are characteristic of both conventional tablets and orodispersible tablets, with a special mention that due to the different release profiles, the conventional tablets exhibit higher values for resistance to crushing, tensile strength and, crushing strength friability ratio, and lower for friability.

**Figure 8:**
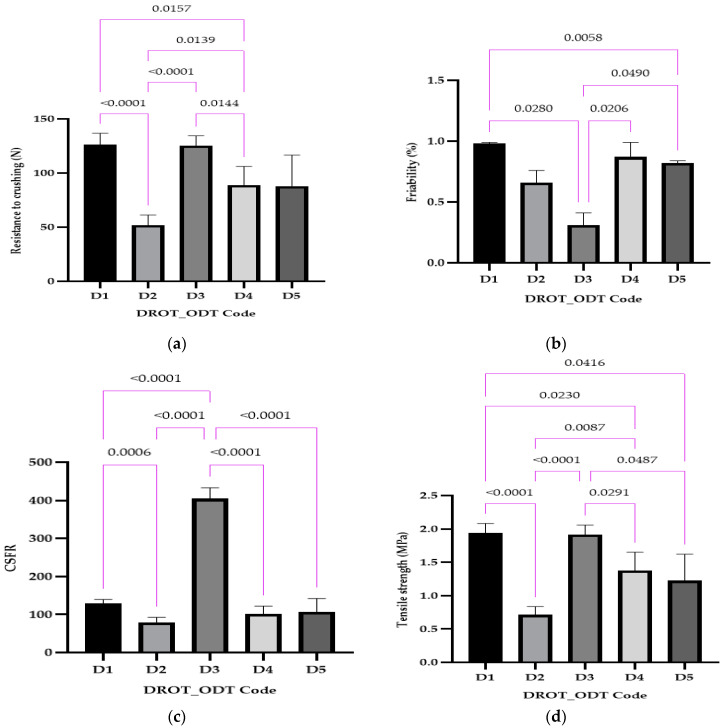
DROT-ODTs resistance to crushing (**a**), friability (**b**), crushing strength-friability ratio (**c**), and tensile strength (**d**) expressed as average ± SD of the developed DROT-ODTs Brown–Forsythe and Welch ANOVA test (multiple comparisons); significance level (*p* < 0.05). D1 (1% EMCS), D2 (5% EMCS), D3 (1% SSP), D4 (5% SSP), and D5 (3% EMCS). All unmarked comparisons correspond to nonsignificant differences (*p* > 0.05).

The resistance to crushing (Figure 8a) varied between 52.00 ± 8.46 N for D2 and 125.50 ± 8.15 N for D3. Statistically significant differences were registered in the case of D1 vs. D2 and D3 vs. D4, where the same disintegrant was used but in different amounts (1% and 5%), the type of disintegrant D2 vs. D4, and the concentration and the type of disintegrant D1 vs. D4 and D2 vs. D3. The unmarked comparisons correspond to differences that were not significant.

Friability was less than 1% for all the evaluated DROT-ODTs, ranging between 0.31% for D3 and 0.98% for D1 (Figure 8b). Statistically significant differences were recorded in the case D1 vs. D3 where 1% of the disintegrant was used but the type of disintegrant was varied, or the concentration of the disintegrant served as a factor for the differences for D1 vs. D5 and D3 and D4, and both of the factors considered for D3 vs. D5.

CSFR ranged between 78.78 for D2 and 404.84 for D3, and the other three formulations outlining a CSFR value between 100 and 130 (D1, D4, D5) (Figure 8c). It can be noticed that in the case of D2, a four-time larger value was obtained, a fact which highlights that D2 has the best mechanical properties and a lower risk of a possible fracture during manipulation. Statistically, the following significant differences emerged: D1 vs. D2 and D3 vs. D4, which can be explained through the different amounts of disintegrant used; D1 vs. D3, explained through the different types of disintegrant; and D2 vs. D3 and D3 vs. D5, explained through both percentage and type of disintegrant.

Tensile strength results (Figure 8d) depict the lowest value for D2 (0.71 MPa) and the highest values for D1 (1.94 MPa) and D3 (1.91 MPa); the other two formulations range in the interval between the minimum and the maximum value. The high value regarding the tensile strength is sustained by the CSFR, friability, and crushing strength, whereas the D3 always had the better values (low friability, high CSFR, and crushing strength). Some differences in statistical significance were recorded in the case of this mechanical parameter: D1 vs. D2, D1 vs. D5, and D3 vs. D4, which can be explained through the different amounts of disintegrant used; D2 vs. D4, explained through the type of disintegrant; and D1 vs. D4, D2 vs. D3, and D3 vs. D5, which can be elucidated through the different amount and type of disintegrant.

Kuralla et al. used the term hardness instead of resistance to crushing and reported values for this parameter between 3 and 4 kg × cm^−2^, which means less than 50 N in comparison to our results in which values larger than 50 N were obtained when developing DROT-ODTs using the solid mixture technique and via melt granulation [2,16]. Furthermore, in both of the studies conducted, Kuralla et al. mentioned friability less than 1%, a value also obtained in this study [2,16]. Regarding the CSFR, it is mentioned that a higher CSFR indicates better mechanical properties [36,37,38]. In comparison with the studies where CSFR was calculated, good results were obtained in our study, indicating proper mechanical properties.

In the study conducted by Brniak and collaborators, tensile strength between 0.7 and 8.25 MPa was obtained, which was increasingly dependent on the compression force. In our study, values less than 2 MPa were obtained, but these results do not imply poor mechanical properties considering the good crushing strength values characteristic of orodispersible tablets [43].

#### 3.3.4. pH

**Figure 9:**
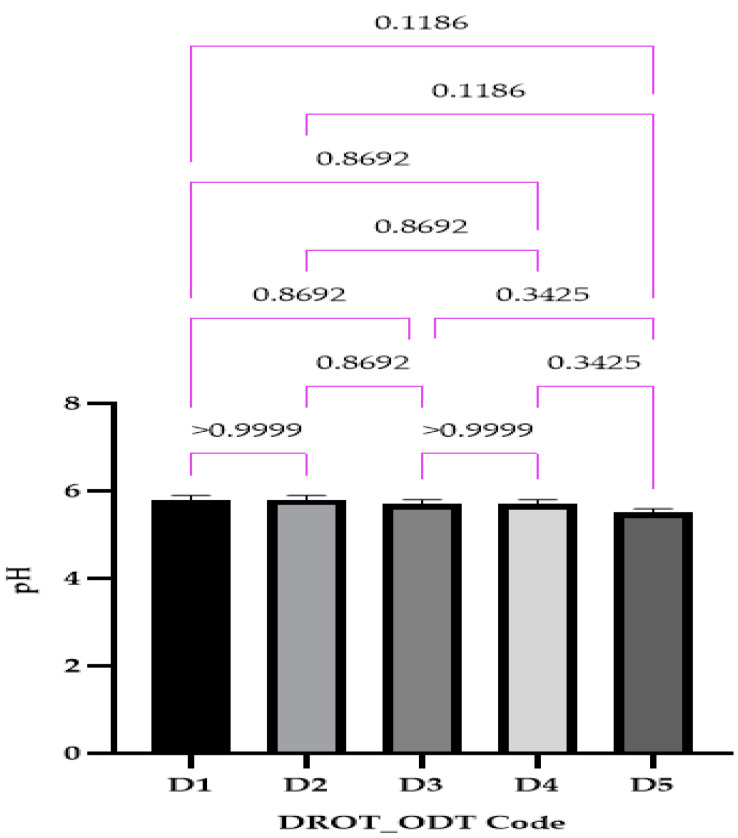
DROT-ODTs pH expressed as average ± SD of the developed DROT-ODTs Brown–Forsythe and Welch ANOVA test (multiple comparisons); significance level (*p* < 0.05). D1 (1% EMCS), D2 (5% EMCS), D3 (1% SSP), D4 (5% SSP), and D5 (3% EMCS).

The pH ranged between 5.5 ± 0.0 for D5 and 5.8 ± 0.0 for D1 (Figure 9). In the case of this parameter, no significant differences were noticed, mainly since the composition differed only through the type of the superdisintegrant used and its solubility in water. Saliva pH ranges betwixt 6.2 to 7.6, so the values obtained will not influence negatively the saliva pH, also their acidic pH might be an advantage due to the possibility of increasing the saliva amount. This parameter is more characteristic of orodispersible films and usually is not evaluated for ODTs.

#### 3.3.5. Wetting Time

**Figure 10:**
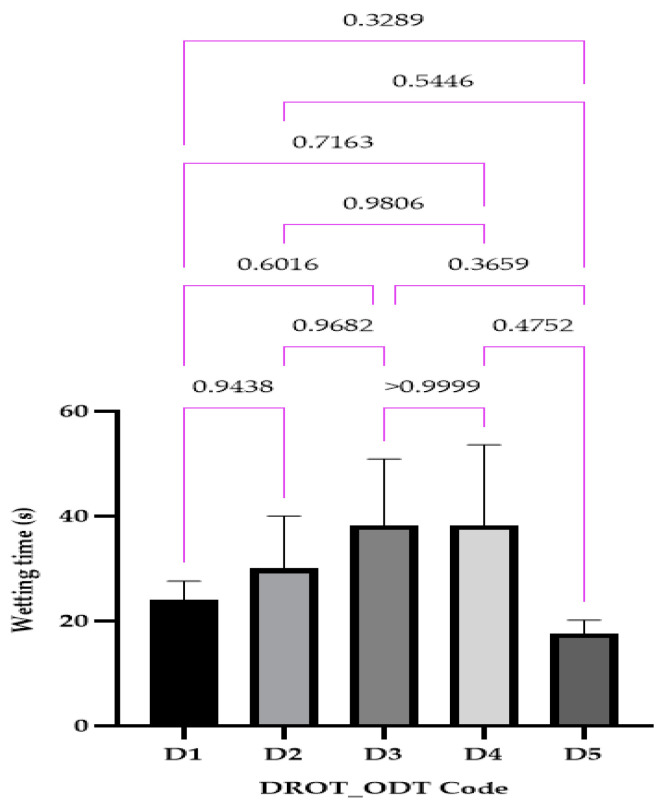
DROT-ODTs wetting time expressed as average ± SD of the developed DROT-ODTs Brown–Forsythe and Welch ANOVA test (multiple comparisons); significance level (*p* < 0.05). D1 (1% EMCS), D2 (5% EMCS), D3 (1% SSP), D4 (5% SSP), and D5 (3% EMCS). All unmarked comparisons correspond to nonsignificant differences (*p* > 0.05).

The wetting time ranged between 17.66 ± 2.05 s (D5) and 38.33 ± 12.47 s for D4 (Figure 10). This parameter can represent a preamble to the disintegration and dissolution behavior, indicating that both of the parameters previously mentioned will be in the pre-establish range obtaining also ODTs with DROT-HCl that will be respecting the fast dissolution requirements.

In the study conducted by Muntean et al., wetting times were between 25.33 s for N8 (mannitol, 4% Crospovidone, no sweetener, and 1% flavor were used) and 542 s for N18 (Isomalt 720, 4% CCNa, 1.5% sweetener, and no flavor were used). The factors responsible for the modifications in this parameter were as follows: increase in Isomalt (ISO); CCNa; disintegrant concentration; sweetener concentration and the interactions between ISO * CCNa; mannitol (MAN * CCNa; Ludipress (LUD) * Crospovidone (CRP); and decrease in MAN; LUD; CRP; flavor amount; and the interactions between ISO * CRP and MAN * CRP [44]. From this article, it can be observed that the type of disintegrant and the percentage of disintegrant are influencing the wetting time [44]. Considering the amounts of disintegrant used in the previously mentioned article, the percentage of disintegrant might influence negatively the wetting time because it reaches a plateau (The maximum amount of disintegrant admitted that produces a fast disintegration was exceeded; as a result, the wetting time is influenced in a negative manner).

In the study where Kuralla et al. developed DROT-ODTs employing a solid mixture, it was observed that the wetting time ranged between 18 s (R1) and 137 s (H1), which was differentiated through the ratio between the active ingredient and the solid mixture, the amount of MAN, the amount of sodium croscarmellose, the amount of magnesium stearate, and the presence/absence of aspartame and PVP K-30. The main factor responsible for these increased differences is the solid mixture and the ratio between the API and the excipient used as a solid mixture [16].

For the same authors’ DROT-ODTs, developed through melt granulation, different wetting times were noted at 24 s (CP9 (where MAN and CCNa were used) and 395 s (CP1, where no disintegrant was used), showing the importance of the disintegrant use and its concentration (8% in the case of CP9) [2].

Another method of developing ODTs was applied by Reddy and collaborators; in this case, different amounts of disintegrant and MAN were used. The wetting time ranged between 26 ± 0.87 s (F5 where 3.92% CCNa and MAN 11.76%) and 175 ± 1.8 s (F1 where 2.27% CCNa and no MAN was used), highlighting the importance of disintegrant concentration and the presence/absence of MAN, a multifaceted excipient that can be used both as a sweetener and as filler [24].

#### 3.3.6. Disintegration Test (Behavior)

**Figure 11:**
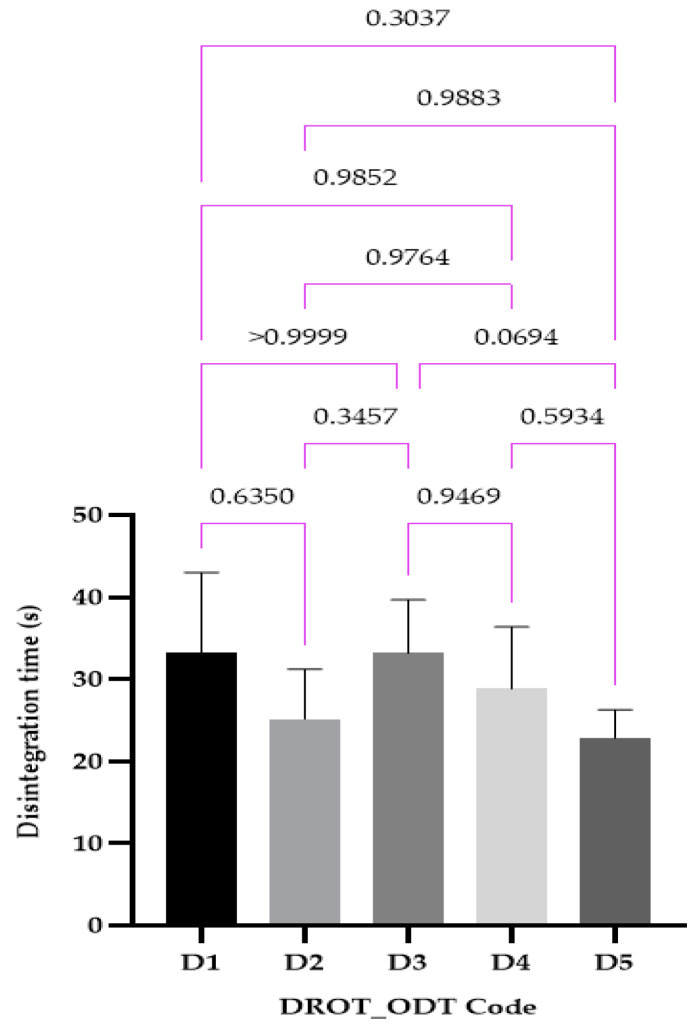
DROT-ODTs disintegration time expressed as average ± SD of the developed DROT-ODTs Brown–Forsythe and Welch ANOVA test (multiple comparisons); significance level (*p* < 0.05). D1 (1% EMCS), D2 (5% EMCS), D3 (1% SSP), D4 (5% SSP), and D5 (3% EMCS). All unmarked comparisons correspond to nonsignificant differences (*p* > 0.05).

The disintegration times were in the range 22.83 ± 3.18 s for D5 and 33.16 ± 9.01 s for the D1 formulation (Figure 11). As in the case of the wetting time, no statistical differences were noticed. Three formulations (D1, D2, D5) respect the strict requirements of the USP 44, whilst all of them present disintegration times that are lower than 180 s, as required by the *Ph. Eur. 10*.

In another study that developed DROT-ODTs through melt granulation, the disintegration times ranged between 32 ± 1.33 s (PF6 5.86% CCNa and 11.72% MAN) and 349 ± 1.04 s (CP1—0% disintegrant and 0% MAN), whilst through the solid mixture technique, the results were between 0.53 ± 1.26 s (H9—3% CCNa and 2% crystalline cellulose) and 90 ± 1.29 s for R7 (1% CCS and 4% PVP—K30). Additionally, in the case of H9, the ratio between the DROT and solid mixture was 1:9, whilst for the R7, it was 1:7.5 [2,16]. Reddy et al. developed DROT-ODTs by using coprocessed excipients. In this study, it was noticed that the disintegration time varied between 45 ± 2.09 s (F5—3.92% CCNa and 11.76% MAN) and 198 ± 1.34 s (F1—2.27% CCNa and no MAN). In this study, the importance of disintegrant percentage and the use of MAN (to improve the disintegration parameter) are highlighted [24].

As can be seen, the type, concentration, or combination of the disintegrants and the lack of disintegrants can influence the disintegration time.

#### 3.3.7. DROT-HCl Content

**Figure 12:**
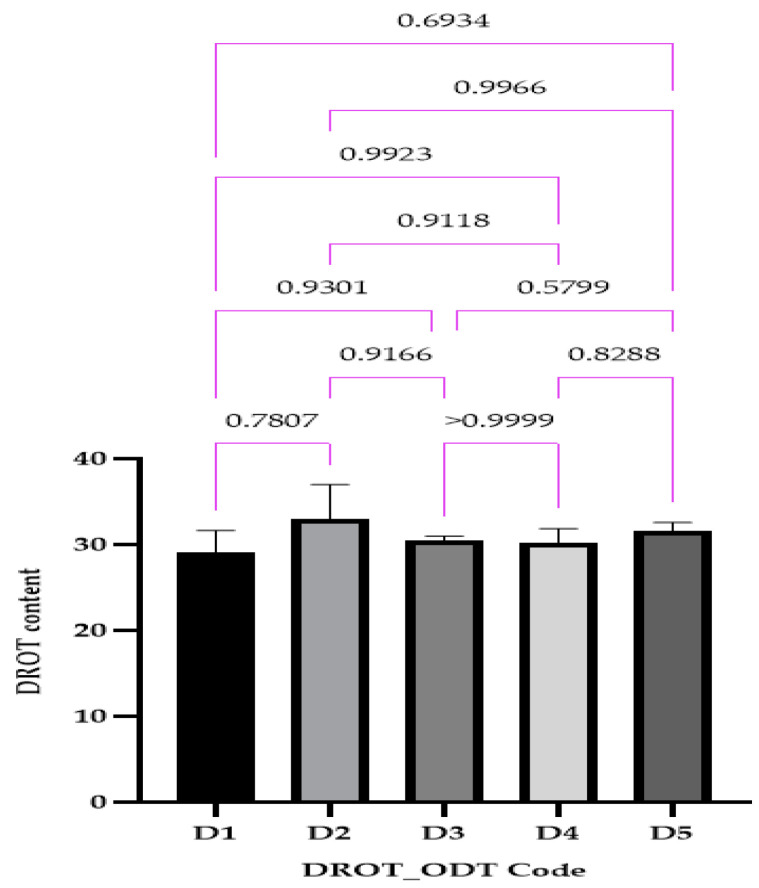
DROT-ODTs content expressed as average ± SD of the developed DROT-ODTs Brown–Forsythe and Welch ANOVA test (multiple comparisons); significance level (*p* < 0.05). D1 (1% EMCS), D2 (5% EMCS), D3 (1% SSP), D4 (5% SSP), and D5 (3% EMCS). All unmarked comparisons correspond to nonsignificant differences (*p* > 0.05).

The DROT-HCl content varied between 29.04 ± 0.85% for D1 and 32.98 ± 1.32% for D2 (Figure 12); for all of the formulation concentrations close to the proposed amount of 30 mg DROT-ODT obtained, no statistical differences were noticed. Kuralla et al. (both studies) and Reddy et al. also obtained good results regarding the uniformity of content, all being in the range 97.79% ± 0.82% and 99.85% ± 0.76% [2,16,24].

#### 3.3.8. Dissolution

**Figure 13:**
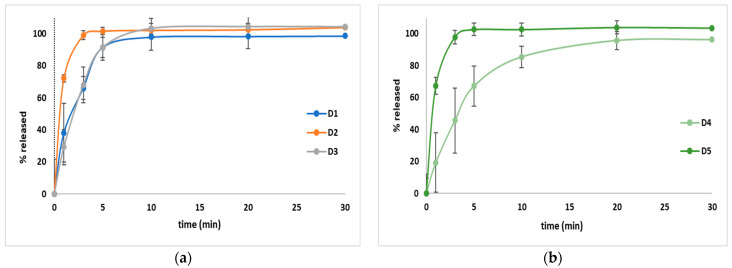
The average amount of DROT-HCl released from the DROT-ODTs developed. D1 (1% EMCS), D2 (5% EMCS), D3 (1% SSP), D4 (5% SSP), and D5 (3% EMCS); (**a**) D1–D3 and (**b**) D4 and D5.

The dissolution behavior is outlined in Figure 13, and this parameter highlights that in the case of all five formulations, over 95% of API was released after 30 min. In the case of D2 and D5, over 95% were released after 3 min, a fact that suggests a high dissolution rate.

To compare our results to the results outlined in other articles, the amount of drug released at 5 min was evaluated. Two studies were taken into consideration due to the similar amount and pH and volume of the dissolution media used [16,24], and it was noticed in both studies that lower amounts of API were released (R1–R11 between 30.7% and 53.5% and H1–H9 between 6.4% and 31.2%), and in the study conducted by Reddy et al., between 8.12% and 62.12% (F1–F6) [24], in comparison to our study where over 90% of the API was released in the case of four formulations (D1–D3 and D5). D4 was an exception, where almost 70% of the API was dissolved. The faster amount of dissolved API can represent an advantage considering the developed pharmaceutical formulation.

## 4. Conclusions

To comply with the fast disintegration time and amounts released, a disintegrant was used in both intragranular (CCSNa) and intergranular (EMCS, SSP) formulations, considering that if a disintegrant would not be used in the granules, the risk of a decrease in the amounts released during the dissolution behavior evaluation might occur. Five formulations of DROT-ODTs were obtained that were evaluated in terms of pharmacotechnical and analytical quality. For all five, the formulations respect the in-force requirements regarding the friability, indicating that this parameter is lower than the one required by the pharmacopeias. The wetting time represents a useful experiment that can profile both the disintegration behavior and the dissolution profile. All of the results regarding the disintegration time respected the maximum admissible value as provided in *Ph. Eur. 10*. The DROT-HCl assay highlighted that none of the formulations suffered from a lack of homogeneity. A fast dissolution profile was set for all five formulations proposed. It was noticed that the selected variables influence the selected responses.

## Figures and Tables

**Table 1:** Patented technologies used in ODT formulations on the market.

Commercial Product	Patented Technology	Active Substance (Dose/Tablet)	Use	Company
Imodium^®^	Zydis ^®^	Loperamide (2 mg)	antidiarrheic	Janssen
Claritine^®^	Zydis ^®^	Loratadine (10 mg)	antihistaminic	Schering Plough
Motilium^®^	Zydis ^®^	Domperidone (10 mg)	antiemetic	Janssen
NuLev^®^	Durasolv^®^	Hyoscyamine sulfate (0.125 mg)	antispasmodic	Alaven
Zomig ZMT^TM^	Durasolv^®^	Zolmitriptan (2.5 or 5 mg)	antimigraine agent	Astra Zeneca
Remeron^®^ SolTab	Orasolv^®^	Mirtazapine (15, 30 or 45 mg)	antidepressant	Organon
Tempra^®^ FirstTabs	Orasolv^®^	Acetaminophen (160 mg)	analgesic	Taisho

**Table 2:** Excipients used in ODTs formulations.

Ingredient Type	Example
Superdisintegrants/disintegrants	Croscarmellose sodium, crospovidone, sodium starch glycolate, sodium carboxymethyl cellulose, sodium alginate
Fillers	Lactose, starch, microcrystalline cellulose, maltodextrins
Sweeteners	Natural sugars (sugar, fructose), sodium saccharin
Lubricants	Magnesium stearate, talc, sodium acetate, stearic acid, Aerosil^®^, liquid paraffin
Emulsifiers	Propylene glycol, sodium lauryl sulfate, polyethylene glycol 4000 and 6000

**Table 3:** DROT-FDGs composition.

Excipient	Quantity (g)
DROT-HCl	10.00
CCSNa	5.10
LCTS	80.08
PVP	1.00
Ethanol 96% (*v*/*v*)	38.04

**Table 4:** The DROT-ODTs composition.

Excipient	Formulation Code				
	D1	D2	D3	D4	D5
	Quantity—*w*/*w* (g)				
DROT-FDGs	0.3816	0.3816	0.3816	0.3816	0.3816
Sucralose	0.05	0.05	0.05	0.05	0.05
Pruv^®^	0.005	0.005	0.005	0.005	0.005
EMCS	0.005	0.025	-	-	0.015
SSP	-	-	0.005	0.025	-
BFL	0.005	0.005	0.005	0.005	0.005
LCTS	0.0534	0.0334	0.0534	0.0334	0.0434
Final mass	0.5000	0.5000	0.5000	0.5000	0.5000

**Table 5:** The cumulative results of the DROT-FDGs particle size distribution.

Sieve Size (µm)	Average Class Interval Sieve Size Xi (µm)	Mass of Granules Retained on the Sieve after 10 min		Cumulative Results	
		g	Ni (%)	N1 (% Retained)	N2 (% Passed)
4000	>4000			0	100
2500	3250	6.85	7.61	7.61	92.93
2500	1650	62.72	69.74	77.35	22.65	
800						
800	557.5	5.97	6.63	83.98	16.02	
315						
315	257.5	1.35	1.50	85.48	14.52	
200						
160	<160	13.04	14.5	99.98	0.02

**Table 6:** The sieve diameter and the corresponding fraction amount used for the calculation of the relative homogeneity index.

Corresponding Diameter	Sieve (mm)	Average Diameter of the Fraction	Corresponding Fraction	Ni (% Retained)
dm+2	4.000–2.500	3.2500	Fm+2	7.61
dm+1	2.500–0.800	1.6500	Fm+1	69.74
dm	0.800–0.315	0.5575	Fm	6.63
dm−1	0.315–0.200	0.2575	Fm−1	1.50
dm−2	0.200–0.160	0.1800	Fm−2	14.5

## Data Availability

On ResearchGate.

## Supplementary Materials

The following supporting information can be downloaded at: https://www.mdpi.com/article/10.3390/pharmaceutics15082147/s1, Figure S1: The DROT-ODGs Appearance; Figure S2: UV-Vis spectra of the drotaverine hydrochloride (a) and the linearity of the spectrophotometric method (concentration range 1-100 µg/mL) (b).

## References

[1] Ronge P.P., Shinde D.A.D. (2019). A review on—Formulation and in-vitro evaluation of orodispersible tablets. Indo Am. J. Pharm. Sci..

[2] Kuralla H., Saripilli R., Kolapalli V.R.M. (2018). Preparation and evaluation of drotaverine hydrochloride orally disintegrating tablets using melt granulation. J. Appl. Pharm. Sci..

[3] Fouad S.A., Malaak F.A., El-Nabarawi M.A., Abu Zeid K. (2020). Development of orally disintegrating tablets containing solid dispersion of a poorly soluble drug for enhanced dissolution: In-vitro optimization/in-vivo evaluation. PLoS ONE.

[4] Deshpande K.B., Ganesh N.S. (2011). Orodispersible tablets: An overview of formulation and technology. Int. J. Pharma Bio Sci..

[5] Kakar S. (2018). Orodispersible tablets: An overview. MOJ Proteom. Bioinform..

[6] Goel H., Vora N., Tiwary A.K., Rana V. (2009). Understanding the mechanism for paradoxical effect of ionized and unionized chitosan: Orodispersible tablets of Ondansetron Hydrochloride. Pharm. Dev. Technol..

[7] Unvala H.M., Schwartz J.B., Schnaare R.L. (1988). The Effect of the Wet Granulation Process on Drug Dissolution. Drug Dev. Ind. Pharm..

[8] Shanmugam S. (2017). Granulation techniques and technologies: Recent progresses. BioImpacts.

[9] Al-khattawi A., Mohammed A.R. (2013). Compressed orally disintegrating tablets: Excipients evolution and formulation strategies. Expert. Opin. Drug Deliv..

[10] Ghourichay M.P., Kiaie S.H., Nokhodchi A., Javadzadeh Y. (2021). Formulation and Quality Control of Orally Disintegrating Tablets (ODTs): Recent Advances and Perspectives. BioMed Res. Int..

[11] Shirsand S., Suresh S., Swamy P., Para M., Nagendra Kumar D. (2010). Formulation design of fast disintegrating tablets using disintegrant blends. Indian J. Pharm. Sci..

[12] Vlad R.A., Antonoaea P., Redai E.M., Muntean D.L., Todoran N., Bîrsan M., Tataru A., Ciurba A. (2021). Soy polysaccharides therapeutic and technological aspects. Acta Marisiensis-Seria Medica.

[13] Chivero P., Gohtani S., Ikeda S., Nakamura A. (2014). The structure of soy soluble polysaccharide in aqueous solution. Food Hydrocoll..

[14] Hosny K., Mosli H., Hassan A. (2015). Soy polysaccharide as a novel superdisintegrant in sildenafil citrate sublingual tablets: Preparation, characterization, and in vivo evaluation. Drug Des. Devel Ther..

[15] Mv S., Mu U., Sa S., Kv R.M. (2010). Design and evaluation of taste masked Drotaverine HCl orodispersible tablets using polymethacrylate polymers. Pharm. Lett..

[16] Kuralla H., Saripilli R., Ramana V., Kolapalli M. (2018). Preparation and evaluation of drotaverine HCl oral disintegration tablets using solid mixture technique. Asian J. Pharm. Clin. Res..

[17] Simon G., Vargay Z., Winter M., Szüts T. (1979). The intestinal absorption and excretion of14C drotaverin in rats. Eur. J. Drug Metab. Pharmacokinet..

[18] Dyderski S., Grześkowiak E., Drobnik L., Szalek E., Balcerkiewicz M., Dubai V. (2011). Bioavailability Study of Drotaverine from Capsule and Tablet Preparations in Healthy Volunteers. Arzneimittelforschung.

[19] Deepshikha, Soni M., Gupta D., Godara S. (2020). Drotaverine hydrochloride versus valethamate bromide for cervical dilatation in labour: A comparative study. Int. J. Reprod. Contracept. Obstet. Gynecol..

[20] Vargay Z., Simon G., Winter M., Szüts T. (1980). Qualitative and quantitative determination of drotaverine metabolites in rat bile. Eur. J. Drug Metab. Pharmacokinet..

[21] Sunita A., Hindumathi M., Sivajyothi I. (2015). The comparative study of drotaverine hydrochloride vs valethamate bromide in labor. J. Evol. Med. Dent. Sci..

[22] Rai R., Dwivedi M., Kumar N. (2014). Efficacy and safety of drotaverine hydrochloride in irritable bowel syndrome: A randomized double-blind placebo-controlled study. Saudi J. Gastroenterol..

[23] Bishal A., Ali K.A., Bandyopadhyay B., Bandyopadhyay R., Debnath B. (2022). Study of different super-disintegrants and their use as a magic ingredient for different immediate- release tablets. J. Pharm. Negat. Results.

[24] Reddy D.N., Pavan J.K., Sravan V.D.C., Reddy V.S., Bhasha S.J. (2018). Preparation and Characterization of Orodispersible tablets of Antispasmodic drug manufactured with Co-Processed mixtures. Int. J. Adv. Pharm. Biol. Chem..

[25] https://nomenclator.anm.ro/medicamente?dci=drotaverinum&page=1.

[26] Aguilar J.E., García Montoya E., Pérez Lozano P., Suñe Negre J.M., Miñarro M., Ticó J.R. (2013). New SeDeM-ODT expert system: An expert system for formulation of orodispersible tablets obtained by direct compression. Formulation Tools for Pharmaceutical Development.

[27] Wan S., Yang R., Zhang H., Li X., Gum M., Guan T., Ren J., Sun H., Dai C. (2019). Application of the SeDeM Expert System in Studies for Direct Compression Suitability on Mixture of Rhodiola Extract and an Excipient. AAPS PharmSciTech.

[28] Zhang Y., Xu B., Wang X., Dai S., Shi X., Qiao Y. (2019). Optimal Selection of Incoming Materials from the Inventory for Achieving the Target Drug Release Profile of High Drug Load Sustained-Release Matrix Tablet. AAPS PharmSciTech.

[29] Aguilar-Díaz J.E., García-Montoya E., Pérez-Lozano P., Suñe-Negre J.M., Miñarro M., Ticó J.R. (2009). The use of the SeDeM Diagram expert system to determine the suitability of diluents-disintegrants for direct compression and their use in formulation of ODT. Eur. J. Pharm. Biopharm..

[30] Aguilar-Díaz J.E., García-Montoya E., Suñé-Negre J.M., Pérez-Lozano P., Miñarro M., Ticó J.R. (2012). Predicting orally disintegrating tablets formulations of ibuprofen tablets. An application of the new SeDeM-ODT expert system. Eur. J. Pharm. Biopharm..

[31] Suñé-Negre J.M., Roig M., Fuster R., Hernández C., Ruhí R., García-Montoya E. (2014). New classification of directly compressible (DC) excipients in function of the SeDeM Diagram Expert System. Int. J. Pharm..

[32] Perez P., Suñé-Negre J.M., Miñarro M., Roig M., Fusterm R., García-Montoya E., Hernándezm C., Ruhi R., Tico J.R. (2006). A new expert systems (SeDeM diagram) for control batch powder formulation and preformulation drug products. Eur. J. Pharm. Biopharm..

[33] Khan A. (2019). Optimization of the process variables of roller compaction, on the basis of granules characteristics (flow, mechanical strength, and disintegration behavior): An application of SeDeM-ODT expert system. Drug Dev. Ind. Pharm..

[34] European Pharmacopoeia (2020). 2.9.5. Uniformity of Mass of Single-Dose Preparations.

[35] Adedokun M.O., Ayorinde J.O., Odeniyi M.A. (2014). Compressional, mechanical and release properties of a novel gum in paracetamol tablet formulations. Curr. Issues Pharm. Med. Sci..

[36] Bamiro O.A., Owodunim A.S., Bakre L.G. (2014). Uwaezuoke O.J. Evaluation of Terminalia randii Baker F. gum as a disintegrant in paracetamol tablet formulation. J. Chem. Pharm. Res..

[37] Kunle O.O., Emeje M. (2019). Starch Source and Its Impact on Pharmaceutical Applications. Chemical Properties of Starch.

[38] (2022). <701> Disintegration. USP-NF.

[39] Vlad R.-A., Antonoaea P., Todoran N., Rédai E.-M., Bîrsan M., Muntean D.-L., Imre S., Hancu G., Farczádi L., Ciurba A. (2022). Development and Evaluation of Cannabidiol Orodispersible Tablets Using a 2^3^-Factorial Design. Pharmaceutics.

[40] Parikh D.M. (2009). Handbook of Pharmaceutical Granulation Technology.

[41] (2001). Pharmaceutics: The Science of Dosage Form Design.

[42] Boschini F., Delaval V., Traina K., Vandewalle N., Lumay G. (2015). Linking flowability and granulometry of lactose powders. Int. J. Pharm..

[43] Brniak W., Jachowicz R., Pelka P. (2015). The practical approach to the evaluation of methods used to determine the disintegration time of orally disintegrating tablets (ODTs). Saudi Pharmaceut. J..

[44] Muntean A.C., Negoi O.I., Rus L.L., Vonica A.L., Tomuță I. (2020). Formulation of orodispersible tablets containing paracetamol and their in vitro characterization—A QbD approach. Farmacia.

