# Supplementary material for: The Influence of the Intergranular Superdisintegrant Performance on New Drotaverine Orodispersible Tablet Formulations
Source: Pharmaceutics. 2023 Aug 16;15(8):2147. doi: 10.3390/pharmaceutics15082147 (PMC10459987; doi:10.3390/pharmaceutics15082147)
Supplement: Supplementary file 1 [file pharmaceutics-15-02147-s001.zip › pharmaceutics-2548286-supplementary.pdf]

Article

The Influence of the Intergranular Superdisintegrant Performance on New Drotaverine Orodispersible Tablet Formulations

Robert-Alexandru Vlad [1,†], Cezara Pintea [2,†], Diana-Andreea Chirteș [3,†], Paula Antonoaea [1,*], Emöke Margit Rédai [1,*], Nicoleta Todoran [1], Magdalena Bîrsan [4] and Adriana Ciurba [1]

[1] Pharmaceutical Technology and Cosmetology Department, Faculty of Pharmacy, George Emil Palade University of Medicine, Pharmacy, Science and Technology of Targu Mures, 540142 Targu Mures, Romania
[2] Faculty of Pharmacy, George Emil Palade University of Medicine, Pharmacy, Science and Technology of Targu Mures, 540142 Targu Mures, Romania
[3] Targu Mures Clinical County Hospital, 540072 Targu Mures, Romania
[4] Drug Industry and Pharmaceutical Biotechnology Department, Faculty of Pharmacy, "Grigore T. Popa" University of Medicine and Pharmacy from Iasi, 700115 Iasi, Romania
* Correspondence: paula.antonoaea@umfst.ro (P.A.); emoke.redai@umfst.ro (E.M.R.)
† These authors contributed equally to this work.

1. Supplementary material:

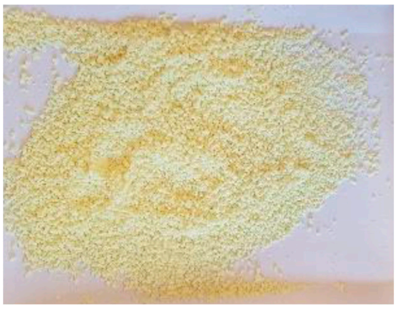

Figure S1. The DROT-ODGs Appearance

2. The spectrophotometric method – analytical performance verification

During the evaluation of the percentage of DROT-HCl released from the ODGs and ODTs the following analytical parameters were verified:

- Linearity: R^2=0.9994, resulting in the equation outlined in Figure S2.a.
- Selectivity: as described below.

It was noticed that in the case of the selected active pharmaceutical ingredient (API) two specific wavelengths were recorded at 308 nm and 353 nm both peaks having the same intensity. The fact that the peaks are in this spectral zone represents an advantage considering that most of the APIs exhibit a peak in the 200-250 nm which is not specific and the risk of interference with other APIs or excipients is increased. To exclude the risk of interference the selectivity of the method was evaluated using a placebo tablet dissolved in the proposed dissolution media. No spectral activity was registered at the selected wavelength.

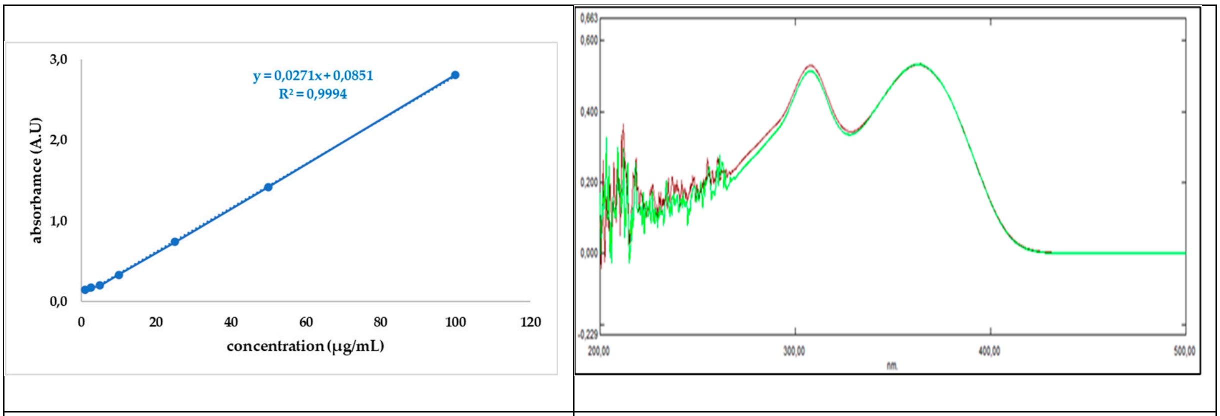

**a)**	**b)**

Figure S2. UV-Vis spectra of the drotaverine hydrochloride (a) and the linearity of the spectrophotometric method (concentration range 1-100 µg/mL) (b)
